# Rapamycin treatment for amyotrophic lateral sclerosis: Protocol for a phase II randomized, double-blind, placebo-controlled, multicenter, clinical trial (RAP-ALS trial)

**DOI:** 10.1097/MD.0000000000011119

**Published:** 2018-06-15

**Authors:** Jessica Mandrioli, Roberto D’Amico, Elisabetta Zucchi, Annalisa Gessani, Nicola Fini, Antonio Fasano, Claudia Caponnetto, Adriano Chiò, Eleonora Dalla Bella, Christian Lunetta, Letizia Mazzini, Kalliopi Marinou, Gianni Sorarù, Sara de Biasi, Domenico Lo Tartaro, Marcello Pinti, Andrea Cossarizza, RAP-ALS investigators group

**Affiliations:** a Department of Neuroscience, St. Agostino-Estense Hospital, Azienda Ospedaliero Universitaria di Modena, University of Modena and Reggio Emilia, Modena; b Unit of Statistics, Department of Diagnostic, Clinical and Public Health Medicine, University of Modena and Reggio Emilia, Azienda Ospedaliero Universitaria di Modena, Modena; c Department of Neurosciences, Rehabilitation Ophthalmology, Genetics, Mother and Child Disease, Ospedale Policlinico San Martino, Genova; d “Rita Levi Montalcini”—Department of Neurosciences, ALS Centre, University of Turin and Azienda Ospedaliero Universitaria Città della Salute e della Scienza, Turin; e 3rd Neurology Unit and ALS Centre, IRCCS “Carlo Besta” Neurological Institute, Milan; f NEuroMuscular Omnicenter, Serena Onlus Foundation, Milan; g ALS Centre, Neurologic Clinic, Maggiore della Carità University Hospital, Novara; h ALS Center, “Salvatore Maugeri” Clinical-Scientific Institutes, Milan; i Department of Neurosciences, University of Padua, Padua,; j Department of Life Sciences, University of Modena and Reggio Emilia, Modena; k Department of Medical and Surgical Sciences for Children and Adults, University of Modena and Reggio Emilia School of Medicine, Modena, Italy.

**Keywords:** amyotrophic lateral sclerosis, autophagy, randomized clinical trial, Rapamycin, Treg lymphocytes

## Abstract

**Introduction:**

Misfolded aggregated proteins and neuroinflammation significantly contribute to amyotrophic lateral sclerosis (ALS) pathogenesis, hence representing therapeutic targets to modify disease expression. Rapamycin inhibits mechanistic target of Rapamycin (mTOR) pathway and enhances autophagy with demonstrated beneficial effects in neurodegeneration in cell line and animal models, improving phenotype in SQSTM1 zebrafish, in *Drosophila* model of ALS-TDP, and in the TDP43 mouse model, in which it reduced neuronal loss and TDP43 inclusions. Rapamycin also expands regulatory T lymphocytes (Treg) and increased Treg levels are associated with slow progression in ALS patients.

Therefore, we planned a randomized clinical trial testing Rapamycin treatment in ALS patients.

**Methods:**

RAP-ALS is a phase II randomized, double-blind, placebo-controlled, multicenter (8 ALS centers in Italy), clinical trial. The primary aim is to assess whether Rapamycin administration increases Tregs number in treated patients compared with control arm. Secondary aims include the assessment of safety and tolerability of Rapamycin in patients with ALS; the minimum dosage to have Rapamycin in cerebrospinal fluid; changes in immunological (activation and homing of T, B, NK cell subpopulations) and inflammatory markers, and on mTOR downstream pathway (S6RP phosphorylation); clinical activity (ALS Functional Rating Scale-Revised, survival, forced vital capacity); and quality of life (ALSAQ40 scale).

**Discussion:**

Rapamycin potentially targets mechanisms at play in ALS (i.e., autophagy and neuroinflammation), with promising preclinical studies. It is an already approved drug, with known pharmacokinetics, already available and therefore with significant possibility of rapid translation to daily clinics. Findings will provide reliable data for further potential trials.

**Ethics and dissemination:**

The study protocol was approved by the Ethics Committee of Azienda Ospedaliero Universitaria of Modena and by the Ethics Committees of participating centers (Eudract n. 2016-002399-28) based on the Helsinki declaration.

## 1 Introduction

Protein aggregates into neurons and glial cells are common features of amyotrophic lateral sclerosis (ALS) pathology, and have a key role in ALS initiation and progression: TDP43 proteinopathy is a hallmark of >95% of sporadic/nonmutated ALS.^[1,2]^ Protein degradation machinery and autophagy have a crucial role in dealing with misfolded aggregated proteins.^[3]^ In ALS several genes (UBQLN2, SQSTM1, OPTN, VCP, TBK1) have strong links with protein degradation pathways as their products contribute to recruitment of ubiquitinated proteins to the autophagosome. Autophagy is also required for the removal of aberrant stress granules involved in ALS pathology ^[4]^ and for downregulation of inflammasome activity, which is activated in response to cellular inclusions formation.^[5]^ TBK1, OPTN, and SQSTM1 converge on autophagy and neuroinflammation, suggesting that compounds which affect both pathways may be promising.^[1]^ A recent paper showed a function for C9orf72 at lysosomes, with impaired responses of mechanistic Target of Rapamycin (mTOR) Complex 1 (mTORC1) signaling to changes in aminoacid availability after depletion of either C9orf72 or SMCR8.^[6]^

mTOR integrates signals to elicit critical outputs, including growth control, protein synthesis, gene expression, and metabolic balance. ^[7]^ The action of Rapamycin is based on mTORC1 inhibition. mTORC1 targets regulatory proteins in cell signaling and regulates autophagy by inhibiting the unc-51-like kinase 1 complex. Inhibition of mTORC1 by Rapamycin stimulates autophagy, through the formation of autophagosome from the phagophore.

mTOR acts on homeostasis of T cells. Indeed, naive CD4+ T cells can develop into TH1, TH2, or TH17 effectors using pathways promoted by mTOR. Conversely, mTOR inhibits the induction of regulatory T lymphocytes (Treg), cells that downregulate immune activation. Inhibition of mTORC1 by Rapamycin expands Tregs and, in mSOD1 mice, increased Tregs and induction of M2 microglia (with anti-inflammatory properties) were associated with stable phase of disease. In ALS patients, blood percentage of Tregs inversely correlated with progression rate, and FoxP3 levels were early predictors of ALS progression and survival.^[8]^ Thus, Tregs may be considered important therapeutic targets in ALS addressed by Rapamycin.

Rapamycin has been tested in several neurodegeneration models because it is an mTOR-dependent autophagy activator, and accelerates the removal of abnormal accumulation of aggregated proteins ^[9,10]^ with beneficial effects.^[7]^ Rapamycin administration exerted a beneficial effect in several cell lines,^[9,11–13]^ and in several ALS animal models, improving motor and cognitive phenotype.^[14–18]^

Based on these premises, we are going to perform a clinical trial in ALS patients with Rapamycin, a drug that enhances autophagy, facilitates TDP43 clearance, and regulates immune responses.

## 2 Methods

### 2.1 Objectives

The primary objective is to assess whether different Rapamycin doses increase Treg number in ALS patients compared with the control arm.

Secondary objectives include the following:

1. *Assessment of Rapamycin safety and tolerability in a cohort of ALS patients*: occurrence of adverse events (AEs), changes on clinical examination including vital signs and weight, and laboratory examinations (biochemistry, hematology and urinalysis) will be registered throughout the entire study duration.
2. *Biological assessment*: To analyze Rapamycin efficacy in inhibiting MTOR pathway, by quantifying the phosphorylation of the S6 ribosomal protein (S6RP) comparing baseline and weeks 8, 18 (treatment end), 30, and 54 between Rapamycin arms and placebo arm. To identify changes in activation and homing capabilities of different T, B, NK cell subpopulations comparing baseline and weeks 8, 18 (treatment end), 30, and 54 between Rapamycin arms and placebo arm. To study Rapamycin effects on different biomarkers (including peripheral and CSF biomarkers, that is, creatinine and albumin, CK, vitamin D, plasma/CSF neurofilament heavy/light chain protein) comparing baseline and week 8, 18 (treatment end), 30, and 54 between Rapamycin arms and placebo arm. To identify Rapamycin-induced changes in inflammatory status, by the molecular analysis of the inflammasome system baseline and week 8, 18 (treatment end), 30, and 54 between Rapamycin arms and placebo arm. To assess Rapamycin capacity to pass through blood–brain barrier (BBB).
3. *Clinical assessment*: to assess changes in Amyotrophic Lateral Sclerosis Functional Rating Scale (ALSFRS)-Revised from baseline to weeks 4, 8, 12, 18, 30, 42, and 54 in placebo and treatment arms; overall survival from randomization to date of documented death or tracheostomy; survival rate at weeks 18, 30, 42, and 54; forced vital capacity (FVC) score from baseline to weeks 4, 8, 12, 18, 30, 42, and 54.
4. Quality of life assessment: ALSAQ-40 from baseline to weeks 8, 18, 30, and 54 in treatment and placebo arms.

### 2.2 Design of the study

This is a multicenter, randomized, double-blind, placebo-controlled, phase II clinical trial. It has been designed following the guidelines on clinical investigation of medicinal products for the treatment of ALS provided by the European Medicines Agency and adopted by the Agenzia Italiana del Farmaco. The protocol has been discussed and revised according to the suggestions of the Data Safety and Monitoring Board (DSMB). The DSMB includes:

- Prof. Lawrence Korngut, University of Toronto.
- Prof. Paola Minghetti, University of Milan.
- Dr Graziella Filippini, IRCCS Foundation “Carlo Besta” Neurological Institute, Milan.
- Dr Ettore Beghi, IRCCS “Mario Negri” Institute, Milan.

The protocol (version 2, April 21, 2017) has been designed adhering to the *Standard Protocol Items for Randomized Trials (SPIRIT)*. The trial has been registered on EU Clinical Trials Register (https://www.clinicaltrialsregister.eu/ctr-search/trial/2016-002399-28/IT) and on Clinicaltrials.gov (https://clinicaltrials.gov/ct2/show/NCT03359538?term=rapamycin&cond=Amyotrophic+Lateral+Sclerosis&rank=1)

**Figure 1:**
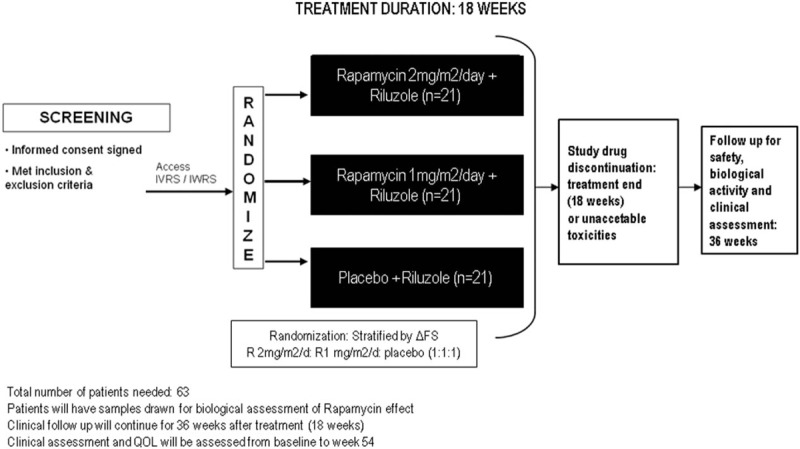
design of the study RAP-ALS.

Figure 1 shows the design of the study.

### 2.3 Eligibility criteria

Patients affected by probable (clinically or laboratory supported) or definite ALS ^[19]^ will undergo screening procedures that must be completed during the 14-day screening period. All patients must adhere to inclusion and exclusion criteria (Table 1) through clinical evaluation and laboratory and instrumental assessment. Screening assessments include blood sampling, biochemical and pregnancy evaluations (for fertile females) which will be performed in the site's local laboratory, general and neurological examinations, MRC, ALSFRS-R, chest radiography and ECG, and spirometry.

**Table 1:**
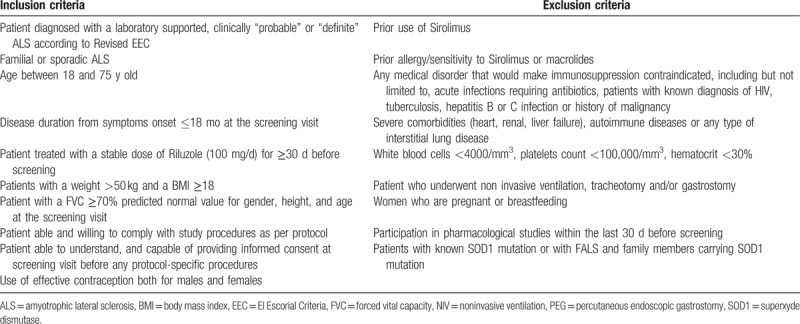
Inclusion and exclusion criteria for RAP-ALS trial.

### 2.4 Randomization

Eligible patients will be randomized strictly sequentially, according to a randomization list prepared by the Biostatistician of the University of Modena.

All patients will receive a unique patient identification number at screening visit when signing the informed consent and before any study procedures are performed. Sixty-three patients will be randomized in the following 3 groups:

1. 21 patients will receive Rapamycin 2 mg/m^2^/d + riluzole.
2. 21 patients will receive Rapamycin 1 mg/m^2^/d + riluzole
3. 21 patients will receive placebo + riluzole

### 2.5 Stratification

Randomization will be performed online. Patients will be stratified according to ΔFS (</≥0.7),^[20]^ with 1:1:1 allocation in 3 treatment arms (Rapamycin 1 mg/m^2^/d; Rapamycin 2 mg/m^2^/d, placebo). The investigators will randomize patients directly online. Treatment will begin within 14 days from randomization. In case of discontinuation from the study, the randomization number will not be reused.

### 2.6 Experimental drug preparation

Pfizer S.r.L. gave the active treatment (Rapamycin) free of charge. Euromed Clinical Supply Services (ECLISSE) (Cantù (Como), Italy; http://www.css.euromed.it/en/) prepared the active formulation and the placebo. The active powder and the investigational drug have been produced complying the Good Manufacturing Practices of the European Union for active pharmaceutical ingredients and ICH Q7A guidelines. Verum and placebo are identical and unrecognizable tablets by form size, weight, and color, and will be indistinguishable to patients and neurologists.

### 2.7 Treatment and blindness

This study is a phase II randomized, double-blind, placebo-controlled, multicenter, clinical trial (RCT) to test Rapamycin in ALS patients. The study will include 63 ALS patients (EL Escorial Revised Criteria, sporadic and familial) with 1:1:1 allocation in 3 groups of 21 subjects each; treatment will be double blinded, and will last 18 weeks. Active treatment will include oral Rapamycin at 1 mg/m^2^/d or 2 mg/m^2^/d.

Rapamycin levels will be regularly measured and revealed only to an independent monitor, who will input Rapamycin levels on a separate eCRF, and on the basis of that value dosing changes may be performed at each treatment dispensation directly through eCRF (and blinded to caring neurologists who will supply bottles identified by a number and corresponding to different blinded doses) to keep Rapamycin levels from 4 to 12 ng/mL. Computerized randomization will be stratified by ΔFS (</≥0.7).

After treatment end (week 18) patients will be followed up for further 36 weeks. This large period of follow-up has the aim to assess potential late side effects of the treatment and to assess whether eventual biological or clinical effects of the treatment with respect to placebo, may last after drug discontinuation.

For the first 10 patients enrolled, clinical and biological safety assessments will be performed every week for the first month, and then every 2 weeks until the end of treatment. For the remaining 53 patients clinical assessments will be performed every 2 weeks for the first month, and then every 4 weeks during treatment duration.

If at either the interim or the final analyses safety concerns will arise, the trial will be stopped and all available data will be reviewed by the Study Steering Committee.

Table 2 shows study procedures to be undertaken.

**Table 2:**
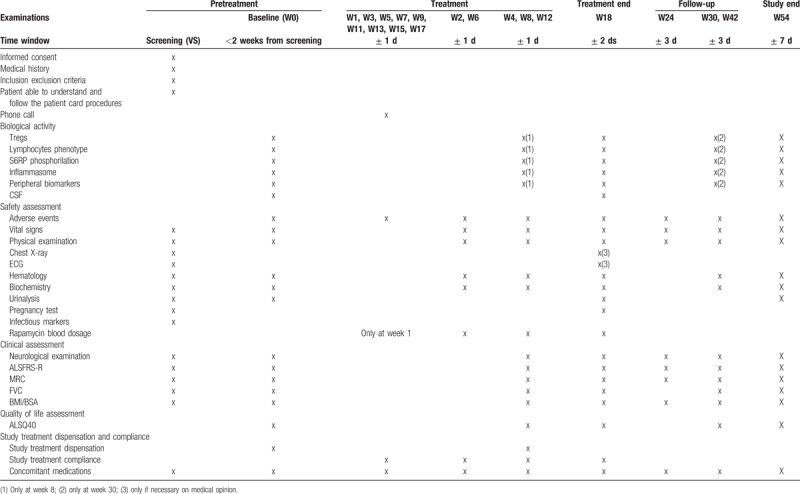
study flowchart (excluding the first 10 patients, for whom additional visits are scheduled).

### 2.8 Concomitant treatments

Patients must have been treated for a minimum of 1 month with a stable dose of riluzole (100 mg/d) at baseline. Safety issues related to riluzole will be managed according to usual practice.

Treatment of ALS impairments (nutrition, respiration, motricity, communication) will be done with a multidisciplinary approach and accordingly to EFNS Guidelines for ALS patients’ management.^[21]^

As for prohibited treatments Rapamycin is known to be a substrate for both cytochrome P-450 3A4 (CYP3A4) and p-glycoprotein (P-gp). Inducers of CYP3A4 and P-gp may decrease Rapamycin concentrations, whereas inhibitors of CYP3A4 and P-gp may increase Rapamycin concentrations. Concomitant use of strong inducers is not recommended, whereas it is forbidden concomitant use of strong inhibitors of CYP3A4 and P-gp.

Alternative agents with lesser interaction potential with Rapamycin should be considered.

Immunosuppressants may affect response to vaccination. Therefore, during treatment with Rapamycin, vaccination may be less effective. Because grapefruit juice inhibits the CYP3A4-mediated metabolism of Rapamycin, it will be not allowed to take with or be used for dilution of Rapamycin.

### 2.9 Outcomes

The primary outcome is represented by the change from baseline to week 18 in Treg number in ALS patients treated with Rapamycin compared with the control arm.

Secondary outcomes are as follows:

1. *Safety*: Occurrence of AEs and per-treatment arising changes in physical examination, vital signs (blood pressure, pulse rate, and body temperature), body weight, and clinical laboratory tests (biochemistry, hematology). MedDRA dictionary is going to be used for reporting.
2. *Rapamycin levels in CSF* at week 18 (high-performance liquid chromatography (HPLC) with mass spectrometry (MS) (LC–MS/MS).
3. Change from baseline to each time point (weeks 8, 18, 30, and 54) of the phosphorylation of the S6 ribosomal protein *(S6RP)* comparing Rapamycin arms and placebo arm.
4. Change from baseline to each time point (week 8, 18, 30, and 54) of the activation and homing capabilities of different *T, B, NK cell subpopulations* comparing Rapamycin arms and placebo arm.
5. Changes from baseline to each time point (weeks 8, 18, 30, and 54) in different *biomarkers* (including peripheral and CSF biomarkers that is, creatinine and albumin, CK, vitamin D, plasma/CSF neurofilament heavy/light chain protein) comparing Rapamycin arms and placebo arm.
6. Changes from baseline to each time point (weeks 8, 18, 30, and 54) in *inflammatory status (molecular analysis of the inflammasome system)* comparing Rapamycin arms and placebo arm.
7. Change from baseline to each time point (weeks 4, 8, 12, 18, 30, 42, and 54) of *ALSFRS-R.*
8. *Survival* defined as the time from randomization to the date of documented death or tracheotomy.
9. Survival rate at weeks 18, 36, and 54.
10. *Change of FVC* from baseline to each time point (weeks 4, 8, 12, 18, 30, 42, and 54).
11. Absolute and relative change from baseline in *ALSAQ-40* (Amyotrophic Lateral Sclerosis Specific Assessment Questionnaire at weeks 4, 8, 12, 18, 30, 42, and 54).

### 2.10 Adverse events

AEs (serious and nonserious AEs), adverse drug reaction (ADR), and unexpected ADR will be defined accordingly to “ICH Guidance for Clinical Safety Data Management: Definitions and Standards for Expedited Reporting.”

All AE occurring between the first study-related procedure and the last study-related procedure will be reported. Those meeting the definition of serious AE (SAE) will be reported in an ad hoc SAE Form; they will be reported to coordinating center within 24 hours.

The following AEs have been reported during the use of Rapamycin, mainly in transplanted patients and in association with other drugs (Table 3).

**Table 3:**
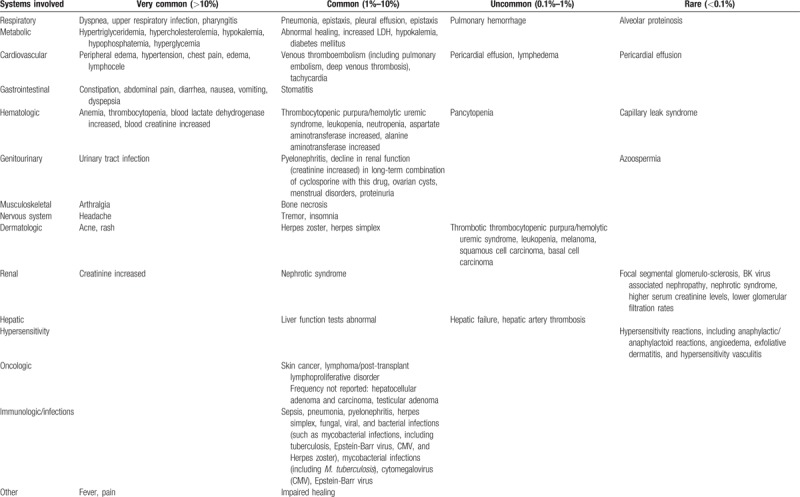
Adverse reactions reported with Rapamycin use.

Investigators must adhere to specific safety rules established for the following toxicities, regardless of the causal relationship to study treatment: respiratory toxicity; metabolic toxicity; cardiovascular toxicity; gastrointestinal toxicity; hematological toxicity; genitourinary toxicity; renal toxicity; reproductive system and pregnancy; hypersensitivity Reactions; skin toxicity; carcinogenicity; and increased susceptibility to infection.

The risk management plan for AEs not described above and suspected to be related to study treatment is as follows:

- At the first occurrence of moderate AE (grade 2): study treatment will be interrupted until AEs has returned to baseline value or mild intensity, then resumed at the same dose level according to local investigator judgment.
- If the same moderate AE reoccurs, study treatment will be interrupted.
- In case of severe AE (grade 3), study treatment will be interrupted until AE has returned to baseline level or mild intensity, then resumed with a dose reduction (1 step).
- In case of life-threatening or disabling AE (grade 4), study treatment must be definitely discontinued.

### 2.11 Safety assessment

Safety will be assessed on the following variables: occurrence of AEs and per-treatment arising changes in physical examination, vital signs (blood pressure, pulse rate, and body temperature), body weight, and clinical laboratory tests (biochemistry, hematology). MedDRA dictionary is going to be used for reporting.

All AEs will be actively collected at each visit, from spontaneous declarations of the patient as well as from oral inquiry and clinical examination. All concomitant medications and/or therapies should be documented in the patient file and reported in the electronic Case Report Form (eCRF).

The following biological parameters will be assessed according to study flow chart (Table 2)—hematology: count of red blood cells, hemoglobin, hematocrit, total white blood cells count, platelet count, and a differential count including neutrophils, lymphocytes, monocytes, eosinophils, and basophils; biochemistry includes urea, creatinine, albumin, protein electrophoresis, AST (SGOT), ALT (SGPT), gamma GT, LDH, cholesterol, triglycerides, sodium, potassium, glucose; screening for infectious diseases including HIV, hepatitis B, hepatitis C, and tuberculosis. An electrocardiogram and chest X-ray (only posterior–anterior view) will be performed at screening. At treatment end (week 18) these exams can be repeated based on clinical opinion of the caring neurologist.

A physical examination including vital signs (systolic and diastolic arterial pressure, heart rate) and body weight will be performed according to the Visit Schedule (Table 2). Check for adequate contraception will be performed at every visit. Respiratory function will be monitored with FVC.

Stopping rules for single patient have been established for each AE.

Stopping rule for the RCT has been established too. We will consider the following list of toxicities as acceptable: peripheral edema, hypertriglyceridemia, hypercholesterolemia, constipation, arthralgia, thrombocytopenia (not severe), anemia (not severe), leukopenia (not severe), rash, hypertension, increased creatinine, abdominal pain, diarrhoea, headache, fever, urinary tract infection, nausea, lymphocele, tachycardia, stomatitis, abnormal healing, increased lactic dehydrogenase, hypokalemia (not severe), diabetes mellitus, epistaxis, ovarian cysts, and menstrual disorders (amenorrhea and menorrhagia).

The study will be stopped by the DSMB in case of >30% of patients experience one of the following side effects: pneumonia, sepsis, venous thromboembolism, thrombotic thrombocytopenic purpura/hemolytic uremic syndrome, severe leukopenia or anemia or thrombocytopenia, bone necrosis, melanoma, and skin cancer. In other words, the trial will be stopped if any of the abovementioned SAEs occurs in ≥6 patients.

As for mild and moderate AEs, the DSMB will be notified if 50% of all subjects report a given mild or moderate severity adverse effect.

### 2.12 Data recording and data monitoring

The investigators will collect data by entering them directly into the trial database, through electronic case report forms ad hoc developed. At the end of the RCT, database will be locked and an expert Statistician will check the database and extract all data for statistical analyses.

The study will be monitored by a certified contract research organization (CRO). A monitor will be in charge of safety data downloads and review on monthly basis (AEs), laboratory data downloads, including Rapamycin levels on fortnightly basis. Data safety and monitoring board (DSMB) meetings will be scheduled (starting when the first 10 patients enrolled would have been treated for 3 months and then when 50% of patients would have completed at least week 8 of treatment and subsequently after every 3 months). All SAEs will be reviewed by the DSMB. As for mild and moderate AEs, the DSMB will be notified if 50% of all subjects report a given mild or moderate severity adverse effect.

### 2.13 Sample size calculation

The sample size has been estimated considering as the primary outcome measure the proportion of positive response (Tregs number increase) at treatment end (18 weeks) in patients treated with Rapamycin versus placebo.

ALS patients have a slight reduction of Treg% (mean±SD: 2.1 ± 0.7) with respect to healthy controls (2.6 ± 0.6) (Treg% calculated on total lymphocytes; normal values of total lymphocytes: 1000–4500/μL; normal values of total Treg: 71.5 ± 17/μL).^[22]^

Slowly progressive ALS patients have a number of Tregs that is equal to healthy controls, whereas fast progressors have 31% fewer Tregs than slowly progressing patients, and Treg % is inversely correlated with the rate of disease progression.^[8]^ These data indicate that ALS patients have 60 ± 17 Treg/μL (fast progressors: 49.3 Treg/μL; slow progressors: 71.5 Treg/μL). As a result, a “positive response” can be considered an increase of the proportion of Tregs by ≥30%. The null hypothesis is that Rapamycin does not increase significantly the proportion of positive responses in treated patients at 18 weeks compared with their baseline stage and to placebo group. The alternative hypothesis is that Rapamycin determines a proportion of positive responses in ≥50% of treated patients compared with a maximum 5% of patients in the placebo group. The study has been designed to reject the null hypothesis with an alpha error of 0.025 (to take into account a multiple comparison with a control arm) and a power of 0.80. For this purpose, a sample of 54 patients randomized in 3 treatment arms would be needed. Considering an average drop out of 15% then a recruitment of 63 patients will be necessary.

### 2.14 Statistical analyses

Separate analyses will be performed in

1. all randomized subjects receiving ≥1 dose of study medication (intention-to-treat population);
2. all randomized subjects excluding protocol deviations (per protocol, PP population).

Descriptive statistics will be performed comparing the 2 groups of Rapamycin treatment and placebo. Continuous variables will be described using mean and standard deviation or median and interquartile range; categorical variables will be described as counts and percentages.

Safety analysis will be performed in all subjects receiving ≥1 dose of the experimental drug. All AEs, SAEs, and AEs leading to treatment discontinuation will be recorded according to ICH Guidelines, listed, and compared in the treatment arms at any follow-up visit and at the end of the study.

Differences in tracheostomy-free survival (Kaplan–Meier method) between the treated groups and placebo group will be compared using the log-rank test. Cox's proportional hazard model would be used to adjust for any possible unbalanced prognostic factors. Statistical significance will be set at .05 level for a 2-tailed test. Missing data will be handled using the last observation carried forward.

Immune response to Rapamycin will be analyzed as the difference in positive response to Rapamycin (mean Tregs increase >30%) between the placebo group and the Rapamycin groups. This will be calculated with the use of Treg data obtained at baseline and at week 18.

We will compare the mean values of S6RP phosphorylation, of different T, B, NK cell subpopulations, of biomarkers, inflammasome, cytokines, comparing baseline and treatment end (18 weeks) between Rapamycin and placebo arm. Mean differences in plasma concentrations from baseline to week 18 in the 2 treatment arms will be calculated and compared using *t* test or Wilcoxon–Mann–Whitney test.

The mean change over time for the same variables as above will be assessed using repeated measures ANOVA, with treatment as between-subjects factor and time as within-subjects factor. Different models will be used, each with a different biomarker of activity as the dependent variable. Models will be adjusted for any unbalanced distribution of the main prognostic factors (e.g., age) between the 2 treatment arms.

### 2.15 Role of participating centers

Our study group includes 8 centers (Turin, Modena, Genoa, Padua, Novara, and 3 centers in Milan) that have a longstanding history on ALS management, and are referral centers for ALS care following >150 patients/y each. They share an established collaboration in the field of ALS, in clinical management of patients, in the realization of clinical trials, genetic, and epidemiological studies. The analysis of biomarkers will be centralized and performed in a renowned laboratory (University of Modena and Reggio Emilia), with large experience in cell analysis and is internationally renowned for the use of flow cytometry for studies on several human diseases. Randomization and statistics will be also centralized to orchestrate the different phases of the project. Statistical Unit of the University of Modena and Reggio Emilia has considerable experience on RCT project and management, and on data analysis. All trial activities will be monitored by a CRO.

Each center is expected

1. to randomize ≥7 patients fulfilling including and excluding criteria in a period of 12 months and to administer the treatment for 18 weeks;
2. to provide 1 principal investigator and 1 neurologist to evaluate including and excluding criteria, get an informed consent, administer treatment, and assess primary and secondary outcome;
3. to provide a person who will send Rapamycin dosage to the coordinating center and who will not see patients; and
4. to formally adhere to the practice parameters of the European Federation of Neurological Societies concerning the standardization of the management of the patient in terms of ventilatory support and nutrition.

### 2.16 Ethics and dissemination

The study will be carried out in accordance with the Declaration of Helsinki, as amended by the 64th WMA General Assembly, Fortaleza, Brazil, October 2013.

The results of the study will be presented during scientific symposia or published in scientific journals only after review and written approval by the involved parties in full respect of the privacy of the participating subjects.

An insurance company will provide insurance coverage for damages emerging from the trial and involving test subjects treated with the test compound.

## 3 Discussion

An urgent unmet need persists for effective therapies in ALS. The absence of reliable biomarker for disease progression and drugs activity is an important target to be addressed too. This is going to be a randomized clinical trial using a drug (Rapamycin) which potentially targets important mechanisms at play in ALS, in particular autophagy and neuroinflammation.

Preliminary data show that in 2 cell lines, inhibition of mTOR by Rapamycin reduced TDP43 fragments accumulation and restored TDP-43 nuclear localization.^[11]^ In murine and in human stem cell-derived neurons and astrocytes with mutant TDP43, autophagy enhancement improved TDP43 clearance and localization and enhanced survival, showing that autophagy induction mitigates neurodegeneration by TDP43 clearance.^[9]^ In cultured neuronal cells expressing mutant ALS-causing FUS, Rapamycin reduced FUS-positive stress granules, as well as neurite fragmentation and cell death in neurons expressing mutant FUS under oxidative stress.^[12]^ In induced pluripotent stem cells derived from ALS patient, activation of autophagic mechanisms with Rapamycin reduced the accumulation of p62.^[13]^

Early Rapamycin administration to a mouse model with FTLD and cytoplasmic TDP43 ubiquinate inclusions (UBIs) rescued learning/memory deficiencies and motor function disorders. This was associated with reduction of neuronal loss and of TDP43 UBIs.^[14]^ Treatment of zebrafish embryos with Rapamycin yielded an amelioration of locomotor phenotype in a SQSTM1 knock-down model.^[15]^

Treatment with Rapamycin of larvae of a *Drosophila* model carrying VAPB (P58S) mutation determined reversal of VAP (P58S) bouton phenotypes, and Rapamycin decreased the severity locomotive defects in a TDP-43/ALS *Drosophila* model.^[16,17]^

Furthermore, inducing autophagy with Rapamycin was shown to improve in vivo pathology of transgenic mice harboring a disease-causing VCP mutation.^[18]^

However, the failure of Rapamycin with aggravation of neuronal death has also been reported in mSOD1 mice. In this study, mSOD1 mice (which reflects the pathogenic mechanisms of SOD1 ALS, whereas the majority of ALS presents TDP43 UBIs) were treated with 2 mg/kg body weight/d, a dosage >50 times higher than that used in clinical practice (usually 2 mg/d), which gives blood concentrations that have well known toxic effects. A further study showed that this effect was due to excessive immunosuppression, as the treatment on ALS mice lacking mature lymphocytes increased ALS survival.^[23]^ However, being the evidences on Rapamycin action mainly on models linked to TDP43 pathology, we will exclude patients carrying SOD1 mutations.

As mTOR has a role in organisms longevity,^[7]^ Rapamycin has been tested to this extent and it resulted to extend the lifespan of various mouse strains.^[24]^ Rapamycin, then, has been shown to protect against several types of neuronal injury (stroke, atherosclerosis, traumatic brain, and spine injury). Recently, it resulted effective in improving respiratory function in lymphangioleiomyomatosis.^[25]^

Rapamycin has a well-known immunosuppressive action: it is used for the cure of transplanted patients, and in clinical trials to test efficacy in autoimmune disorders, CNS tumors, and other neurological disorders (such as tuberous sclerosis).

As for the role of immunity in ALS, recent studies suggest that innate and adaptive immune systems contribute to ALS progression: Msod1 Tregs cocultured with activated Msod1 microglia attenuated the expression of microglial toxic factors by IL4 release, and promoted MN survival by suppressing M1 activation, inducing an M2 protective phenotype, and reducing the release of ROS. Tregs passive transfer into ALS mice prolonged survival, and FoxP3 mRNA in mSOD1 mice spinal cord decreased with disease progression.^[8]^ In patients, blood Treg percentage inversely correlated with ALS progression rate, and FoxP3 levels were early predictors of ALS progression and survival. These data were confirmed in postmortem studies.^[8]^ Inhibiting the mTOR pathway, Rapamycin induces de novo FoxP3 expression and expands Treg.

The entity of the increase of Tregs induced by Rapamycin is completely unknown in ALS patients. For this reason, we are planning this first trial focused on showing immunological targets engagement by Rapamycin in ALS patients. Moreover, given its large natural structure, there was initial concern that Rapamycin might not be able to cross the BBB; however, in one study sufficient levels of Rapamycin could be found in brain tumors when administered at therapeutic doses.^[26]^ To further address this concern, we are planning to test Rapamycin titer in CSF at treatment end with LC–MS/MS (using HPLC). This procedure will also allow to understand if Rapamycin action depends on BBB crossing or on its action on Treg cells.

In conclusion, the study will give extensive inference to a possible effect of Rapamycin on biomarkers of disease progression. Rapamycin is an already approved drug, with known pharmacokinetics, which is available and therefore there is significant possibility of transferability to patients, and of a rapid translation to daily clinics.

This short study will optimize time and resources to get reliable information for a following RCT to test Rapamycin clinical efficacy in ALS.

## Acknowledgments

The authors acknowledge RAP-ALS investigators group:

Department of Neuroscience, St. Agostino Estense Hospital, Azienda Ospedaliero Universitaria di Modena, Modena, Italy (J. Mandrioli, N. Fini, A. Fasano, E. Zucchi, A. Gessani, P. Nichelli)

Unit of Statistics, Department of Diagnostic, Clinical and Public Health Medicine, University of Modena and Reggio Emilia, Modena, Italy (R. D’Amico, R. Vicini)

Department of Neurosciences, Rehabilitation Ophthalmology, Genetics, Mother and Child Disease, Ospedale Policlinico San Martino, Genova, Italy (C. Caponnetto, C. Cabona)

“Rita Levi Montalcini” Department of Neurosciences, ALS Centre, University of Turin and Azienda Ospedaliero Universitaria Città della Salute e della Scienza, Turin, Italy (A. Chiò, A. Calvo, C. Moglia, U. Manera, G. Fuda, A. Canosa, A. Ilardi)

3rd Neurology Unit and ALS Centre, IRCCS “Carlo Besta” Neurological Institute, Milan, Italy (G. Lauria, E. Dalla Bella)

NEuroMuscular Omnicenter, Serena Onlus Foundation, Milan, Italy (C. Lunetta, F. Gerardi)

ALS Centre, Neurologic Clinic, Maggiore della Carità University Hospital, Novara, Italy (L. Mazzini, A. Scognamiglio, F. De Marchi)

ALS Center, “Salvatore Maugeri” Clinical-Scientific Institutes, Milan, Italy (G. Mora, K. Marinou)

Department of Neurosciences, University of Padua, Padua, Italy (G. Sorarù, M. Gizzi)

Department of Life Sciences, University of Modena and Reggio Emilia, Modena, Italy (M. Pinti, S. De Biasi)

Department of Medical and Surgical Sciences for Children and Adults, University of Modena and Reggio Emilia School of Medicine, Modena, Italy (A. Cossarizza, D. Lo Tartaro)

## Author contributions

**Conceptualization:** JESSICA MANDRIOLI, Roberto D’Amico, Claudia Caponnetto, Adriano Chiò, Sara De Biasi, Marcello Pinti, Andrea Cossarizza.

**Data curation:** JESSICA MANDRIOLI, Roberto D’Amico, Elisabetta Zucchi, Annalisa Gessani, Nicola Fini, Antonio Fasano, Claudia Caponnetto, Adriano Chiò, Eleonora Dalla Bella, Christian Lunetta, Letizia Mazzini, Kalliopi Marinou, Gianni Sorarù, Sara De Biasi, Domenico Lo Tartaro, Marcello Pinti.

**Formal analysis:** JESSICA MANDRIOLI, Marcello Pinti, Andrea Cossarizza.

**Funding acquisition:** JESSICA MANDRIOLI, Roberto D’Amico, Marcello Pinti, Andrea Cossarizza.

**Investigation:** JESSICA MANDRIOLI, Elisabetta Zucchi, Annalisa Gessani, Nicola Fini, Antonio Fasano, Claudia Caponnetto, Adriano Chiò, Eleonora Dalla Bella, Christian Lunetta, Letizia Mazzini, Kalliopi Marinou, Gianni Sorarù, Domenico Lo Tartaro.

**Methodology:** JESSICA MANDRIOLI, Roberto D’Amico, Sara De Biasi, Domenico Lo Tartaro, Marcello Pinti, Andrea Cossarizza.

**Project administration:** JESSICA MANDRIOLI, Andrea Cossarizza.

**Resources:** JESSICA MANDRIOLI, Andrea Cossarizza.

**Software:** Roberto D’Amico.

**Supervision:** JESSICA MANDRIOLI, Adriano Chiò, Marcello Pinti, Andrea Cossarizza.

**Validation:** JESSICA MANDRIOLI.

**Visualization:** JESSICA MANDRIOLI, Andrea Cossarizza.

**Writing – original draft:** JESSICA MANDRIOLI, Elisabetta Zucchi, Annalisa Gessani, Nicola Fini, Sara De Biasi, Domenico Lo Tartaro.

**Writing – review and editing:** Roberto D’Amico, Claudia Caponnetto, Adriano Chiò, Eleonora Dalla Bella, Christian Lunetta, Letizia Mazzini, Kalliopi Marinou, Gianni Sorarù, Marcello Pinti, Andrea Cossarizza.
